# Laser-induced porous graphene electrodes from polyketimine membranes for paracetamol sensing

**DOI:** 10.1098/rsos.230294

**Published:** 2023-08-02

**Authors:** Sabrine Baachaoui, Walid Mabrouk, Khaled Charradi, Bechir Slimi, Ahmed M. Ramadan, Rehab M. I. Elsamra, Akram Alhussein, Sherif M. A. S. Keshk, Noureddine Raouafi

**Affiliations:** ^1^ Faculty of Sciences, Department of Chemistry, University of Tunis El Manar, Campus universitaire de Tunis El Manar, Tunis 2092, Tunisia; ^2^ Laboratory Water, Membranes and Biotechnology of the Environment, Water Research and Technologies Center, Technopark Borj Cedria, Soliman 8020, Tunisia; ^3^ Nanomaterials and Systems for Renewable Energy Laboratory, Research and Technology Center of Energy, Technopark Borj Cedria, Soliman 8020, Tunisia; ^4^ Faculty of Science, Department of Chemistry, Alexandria University, PO Box 426, Alexandria 21321, Egypt; ^5^ Technological Pole of South Champagne, University of Technology of Troyes, Lavoisier Rd., Nogent 52800, France; ^6^ Become: Technology, Science, AI & Automation Lab, 63 rue de Tolbiac, Paris 75013, France

**Keywords:** polyketimine, membrane, laser-induced electrode, sensing, paracetamol, health

## Abstract

The development of cost-effective materials for fabricating electrodes is crucial for drug, pharmaceutical and environmental applications. This paper presents the synthesis and characterization of a novel polyketimine (PKI) membrane obtained by condensing partially of different weight percentages of oxidized polyvinyl alcohol and aminated polyether sulfone. Using the PKI membrane as a scaffold, we introduced laser-induced graphene electrodes (LIGEs) for the efficient electrochemical sensing of paracetamol (PCM), which serves as a model drug. Electrochemical measurements were conducted to assess the physico-chemical properties, including laser-induced porous graphene features, such as the heterogeneous electron transfer (HET) rate and electrochemically active surface area (ECSA). The obtained results demonstrate that the LIGEs exhibit excellent performance in PCM sensing, showing a linear detection range of 50–600 µM with a detection limit (LOD) as low as 14.3 µM and a good selectivity toward uric acid. Furthermore, the functionalization of the electrode surface with AuNPs improved the electrode physico-chemical properties (HET and ECSA) and lowered the detection limit down to 1.1 µM. Consequently, these affordable electrodes hold great potential for analysing other drugs and detecting heavy metal cations in various applications.

## 1. Introduction

Analgesics, such as paracetamol (PCM), are widely used for pain relief and fever reduction. PCM is particularly important because of its effectiveness in various analgesic products, including arthritis treatments, menstrual cramps, neuralgia and high body temperature [1]. While PCM is generally safe when used in moderation (up to 4 grams per day), prolonged and excessive usage can lead to kidney and liver damage owing to the accumulation of harmful metabolites such as *N*-acetyl-p-benzoquinone imine and p-benzoquinone imine [2–4]. Therefore, there is a need for quick, easy and precise methods to detect PCM in pharmaceutical formulations and biological fluids based on cost-effective and disposable electrodes [5,6].

Traditional detection methods, such as mass spectrophotometry, titration, electrophoresis flow injection and liquid chromatography, are sensitive and reliable and often require complex pre-treatment processes, expensive equipment and regular investigations [7–10]. By contrast, electrochemical sensing offers a cost-effective, portable and sensitive solution that meets the WHO ASSURED criteria (i.e. affordable, sensitive, specific, user-friendly, rapid, and robust, equipment-free and deliverable to end-users) [11–14]. The electrochemical approach demonstrates exceptional utility in demanding environments, including war zones, regions affected by natural disasters and low-resource settings [15,16].

The success of electrochemical sensing depends on the choice of electrode materials, including metals, metal oxides, conducting polymers and carbon nanostructures [17]. Recently, laser-induced graphene (LIG) has gained attention as a promising electrode material for many applications [16–20]. Various polymer materials, such as polyimide (PI), polyetherimide (PEI), polyether sulfone (PES) and polyphenyl sulfone (PPSU), as well as biomass-derived materials like cellulose, cork and cloth, have also been explored for this purpose [21–23]. In PI polymers, laser scribing generates high temperatures (above 1000°C) that break the C–O, C=O and C–N chemical bonds, leading to the formation of N-doped graphene sheets through the recombination of carbon and nitrogen atoms in the graphene honeycomb structures [21]. The rapid release of nitric oxide, carbon dioxide and water gases during this process results in the development of three-dimensional porous structures [18]. However, to create sensors with excellent stretchability, post-processing steps are required after the production of porous graphene. This involves laser patterning the created porous graphene from a thick, stretchable substrate onto the top of a hard PI film [24]. While this transfer technique is effective, it imposes limitations on fabricating strain sensors with large surface areas and intricate patterns [24].

Previous studies have explored the use of LIG devices in various applications such as energy storage devices, strain sensors, chemical sensors and biosensors [18–20]. However, there have only been a few instances of using LIG on polymeric membranes for detecting PCM. For instance, Berni *et al.* [25] and Ghanam *et al.* [26] used LIG prepared from the lasing of PI membrane to develop sensitive sensors for PCM detection. Kavai *et al.* fabricated LIG on the surface of PEI to simultaneously detect hydroquinone, PCM and methylparaben [27]. Nasraoui *et al.* modified LIG with multi-walled carbon nanotubes (MWCNTs) and conductive polyaniline (PANI) polymer for sensitive sensing of PCM [28].

In this study, we present a novel approach for fabricating conductive sensors by using electrodes with large surface areas and laser scribing to create graphene from a polyketimine (PKI) film, which is derived from the condensation of OPVA and aminated PES. We specifically focus on the application of these sensors for detecting PCM, as a model drug. To ensure optimal performance, we conducted thorough optimization of the LIG electrode production process and characterized the physico-chemical properties of the electrodes, including HET rate and ECSA. Moreover, we enhanced the physicochemical properties of the electrodes by functionalizing them with gold nanoparticles, resulting in improved sensing capabilities for PCM and a lowered detection limit. Moreover, to gain insights into the structure, composition and surface morphology of the Schiff base films, we employed a range of analytical techniques, including DFT, FTIR, ^1^H-NMR, X-ray diffraction (XRD) and thermogravimetric analysis. This work showcases the immense potential of LIG-based electrochemical sensors for diverse applications. By developing a simple yet effective method for fabricating highly conductive sensors and enhancing their properties through functionalization, we opened up new possibilities in the field of sensor technology.

## 2. Material and methods

### 2.1. Chemicals

All chemicals were purchased from Sigma-Aldrich and were used without further purification. The starting products (OPVA and H_2_N-PES) were synthesized following the methods described in a previous report [29]. All electrochemical experiments were conducted at room temperature (RT) unless otherwise stated.

### 2.2. Preparation of polyketimine

**Figure 1.**
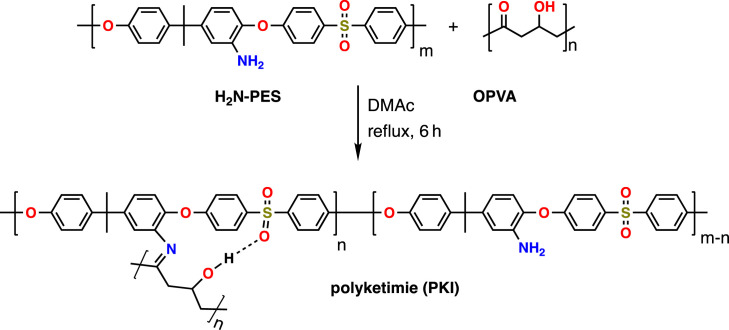
Schematic of the synthesis of PKI through the condensation of H_2_N-PES and OPVA.

To a solution of OPVA (0.58 g, 0.01 mol) in DMAc (20 mL), H_2_N-PES (4.46 g, 0.01 mol) was added. The reaction mixture was heated under reflux for 6 h (figure 1). The yellow product (95% yield) was washed with double distilled water and dried at 60°C overnight.

### 2.3. Physico-chemical characterization

The FT-IR spectra were recorded in transmission mode as a function of wavelength using a Nicolet spectrophotometer (IR 200 FT-IR) in the wavelength range of 400–4000 cm^–1^. NMR spectra (^1^H, 300 MHz) were recorded at RT in DMSO using a Bruker Advance 300 spectrometer. Chemical shifts were expressed in ppm, using tetramethylsilane (TMS) as an internal reference. XRD was measured using a Shimadzu Lab X 6000 X-ray diffractometer with CuK*α* = 1.54 Å radiation and a secondary monochromator at RT. The samples were analysed as compressed tablets for the polymers. The LIG electrodes were used directly without any pretreatment. The XRD tube was operated at 30 kV and 30 mA, with 2*θ* ranging from 10 to 60°. The LIG electrodes were scribed using a 40 W-laser machine equipped with a CO_2_ laser source (*λ* = 10.6 µm) [30]. The power and speed were set to 3.0 W and 150 mm s^–1^, respectively.

Electrochemical measurements were performed using a PC-controlled Palmsens 4 potentiostat equipped with an FRA module. Data were collected using the PS Trace software v. 5.10. The laser-induced electrodes were examined by cyclic voltammetry (CV) and impedance spectroscopy in phosphate-buffered saline (PBS) solution (pH = 7.4). All stock solutions and dilutions were prepared in deionized water.

### 2.4. DFT simulations

In silico analysis of the aminated PES, OPVA and their synthesized PKI was performed using density functional theory (DFT) with the hybrid B3LYP exchange-correlation function combined with a 6–311 + G basis set. This level of theory is a reliable method for calculating the stable closed-shell molecules and H-bonded ligands. The molecular and thermodynamic parameters of the optimized conformers were extracted from the optimization-frequency calculations using Gaussian09 software [31] and visualized using the GaussView package [32].

### 2.5. Fabrication of laser-induced graphene electrodes

The as-prepared film was affixed to the surface of the polyethylene terephthalate (PET) film and dried at RT for 24 h. A three-electrode configuration was scribed using a CO_2_ laser machine. The diameter of the working surface electrode was 0.5 cm, and the connectors were covered with a UV-curable polymer. The laser-induced graphene electrodes (LIGEs) were characterized using CV and electrochemical impedance spectroscopy. Square-wave voltammetry (SWV) was performed to detect PCM. The optimal parameters included a potential amplitude of 10 mV, potential step of 50 mV and frequency of 25 Hz.

**Figure 2.**
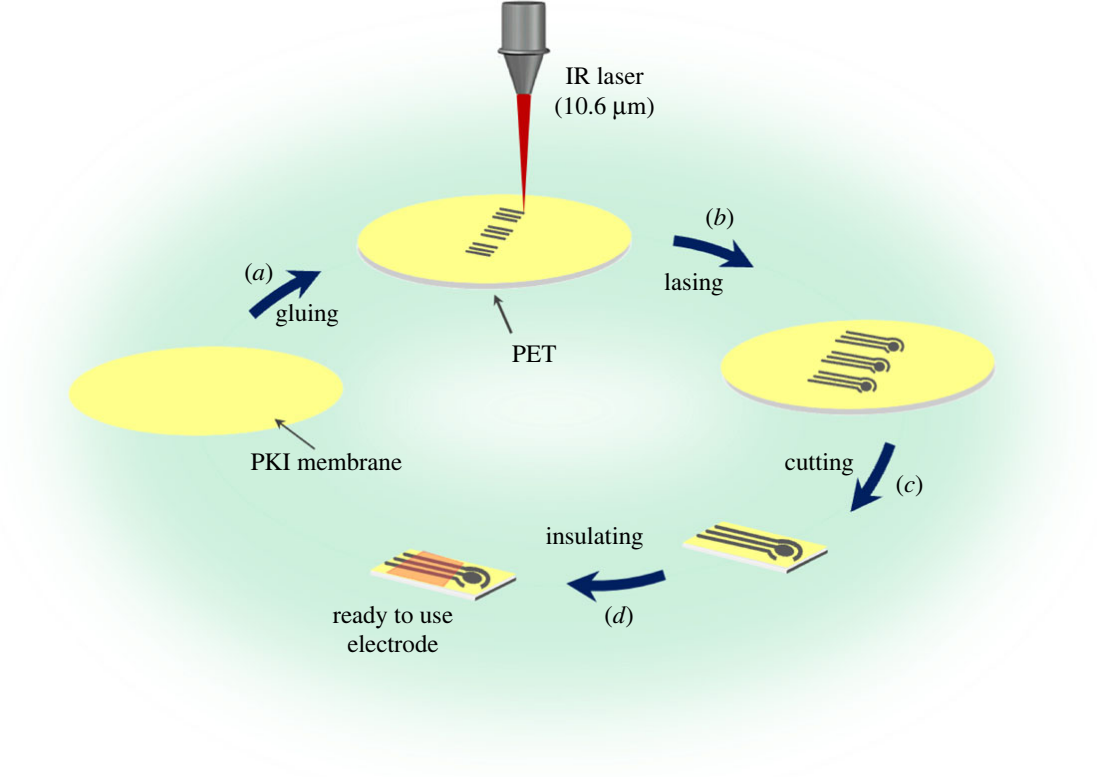
Schematic of the process for preparing laser-induced graphene electrodes: (*a*) polymer affixing on the PET surface, (*b*) laser scribing, (*c*) electrode cutting, and (*d*) insulation with UV-curable polymers.

The overall electrode preparation process is shown in figure 2.

First, the PKI polymer membrane was glued to the PET film to improve its mechanical stability and withstand laser heating up to 1000°C (figure 2*a*). A three-electrode configuration was scribed using a CO_2_ laser system (figure 2*b*). Finally, the electrode was cut, and the connectors were insulated using a non-conducting polymer (figure 2*c* and *d*). The power and speed were optimized to avoid complete burning of the electrode materials [30]. Before use, the electrodes were then cycled in phosphate buffer solution (PBS; pH 7.4) for 20 cycles at a scan rate of 100 mV s^–1^ in order to stabilize their surfaces.

### 2.6. Electrochemistry

#### 2.6.1. Electrochemical measurements

Electrochemical measurements were performed using a PC-controlled Palmsens 4 potentiostat, which was equipped with a frequency response analyzer (FRA) module for conducting electrochemical impedance spectroscopy investigations. All CV and SWV data were collected in a 0.1 M PBS solution containing 5.0 mM concentration of [Fe(CN)_6_]^3–^/[Fe(CN)_6_]^4–^, which served as the redox probe for evaluating electrode surface characteristics. To carry out the electrochemical measurements, PCM and uric acid (UA) stocks were dissolved in a 0.1 M PBS solution, which served as the electrolyte.

For the SWV measurements, the following optimized parameters were employed: a start potential of 100 mV, an end potential of +900 mV, a pulse amplitude of 50 mV, a frequency of 5 Hz and a step potential of 10 mV.

#### 2.6.2. LIGE surface nanostructuration

The functionalization process with AuNPs was carried out in two steps. Firstly, a 50 µL solution of AuNPs, prepared according to the protocols in the literature [33], was applied to the working electrode. The electrode was then dried in an oven at 60°C for 15 min to facilitate the binding of the gold nanoparticles. Following that, a 50 µL solution of H_2_SO_4_ (0.5 M) was applied to the AuNPs-modified electrode, and a potential sweep was carried out from +1.0 V to −0.5 V for 15 cycles at a scan rate of 50 mV s^−1^. This step aimed to activate the electrode surface and enhance the attachment of the nanoparticles. Before using the electrode in further experiments, it was thoroughly rinsed with deionized water to remove any residual substances or impurities.

## 3. Results and discussion

### 3.1. Physico-chemical characterization

The reaction between the amine groups in aminated polyether sulfone and the ketone group in OPVA via condensation to form PKI was confirmed by conducting physico-chemical analyses, as described below.

#### 3.1.1. FTIR spectroscopy

**Figure 3.**
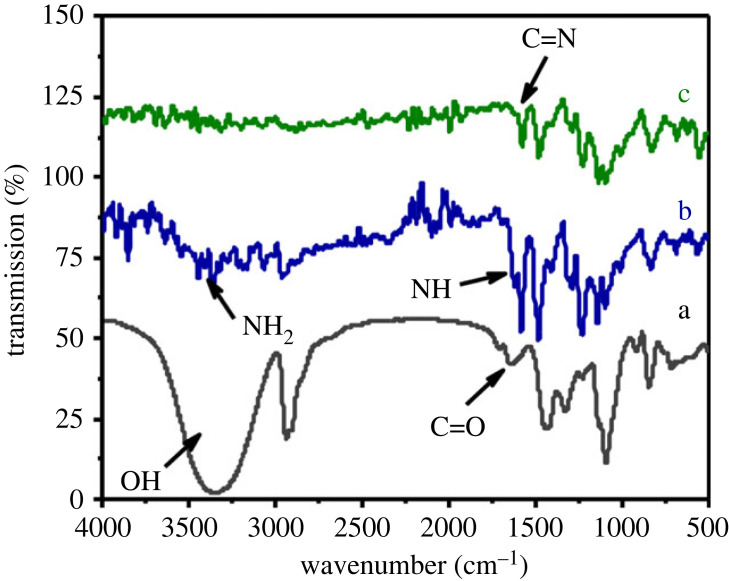
FT-IR spectra of (a) OPVA, (b) H_2_N-PES and (c) PKI.

FTIR spectroscopy was performed to examine the H_2_N-PES, OPVA and PKI polymers at the molecular level (figure 3). The FTIR spectra showed that the major band of OPVA was at 1661 cm^–1^ and was assigned to the carbonyl group stretching vibration band [34]. The primary amine characteristic of H_2_N-PES showed two major stretching vibration bands due to the asymmetric and symmetric stretching vibrations of the N–H group situated from the 3408 cm^–1^ to 3314 cm^–1^ region and one band due to the in-plane bending vibration of the N–H group at 1582 cm^–1^ [35]. In the spectrum of Schiff's base, an absorption band was detected at 1657 cm^–1^, characteristic of the C=N group. This finding confirms the formation of PKI [35].

#### 3.1.2. Proton NMR spectroscopy

**Figure 4.**
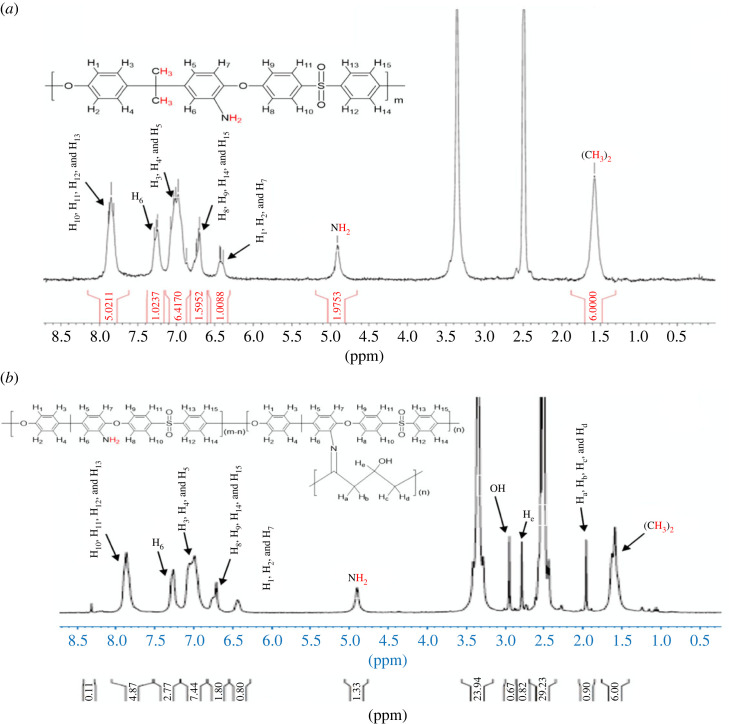
Proton NMR of (*a*) H_2_N-PES and (*b*) PKI.

The chemical structures of the starting amine and its PKI were identified by proton nuclear magnetic resonance (NMR) (figure 2). As shown in figure 4*a*, the ^1^H-NMR spectrum of NH_2_-PES has six characteristic peaks in the aromatic ranges at 6.4, 6.8, 7.0, 7.3 and 7.9 ppm corresponding to protons (H_1_, H_2_, H_7_), (H_8_, H_9_, H_14_, H_15_), (H_3_, H_4_, H_5_), H_6_, (H_10_, H_11_, H_12_) and H_13_, respectively [36]. The protons, amine and dimethyl groups were noted at 4.8 and 1.6 ppm with the integration of approximately 2 and 6, respectively (figure 4*a*).

The integration value of the amine group in NH_2_-PES decreased after the formation of PKI, and the aromatic proton peaks shifted upfield (from 6.5 to 8.2 ppm) compared to the peaks of H_2_N-PES (figure 4*b*) [36]. The characteristic peaks of OPVA present at 2.7–2.9 ppm corresponded to the proton signal of CH_2_CO in the segments, while that of the CH_2_ in the PVA segments overlapped with CH_2_ of NH_2_-PES at 1.5 ppm (figure 4) [37]. These results indicate that OPVA partially reacted with NH_2_-PES to form a PKI polymer.

#### 3.1.3. Modelling the structural stability of PKI

**Figure 5.**
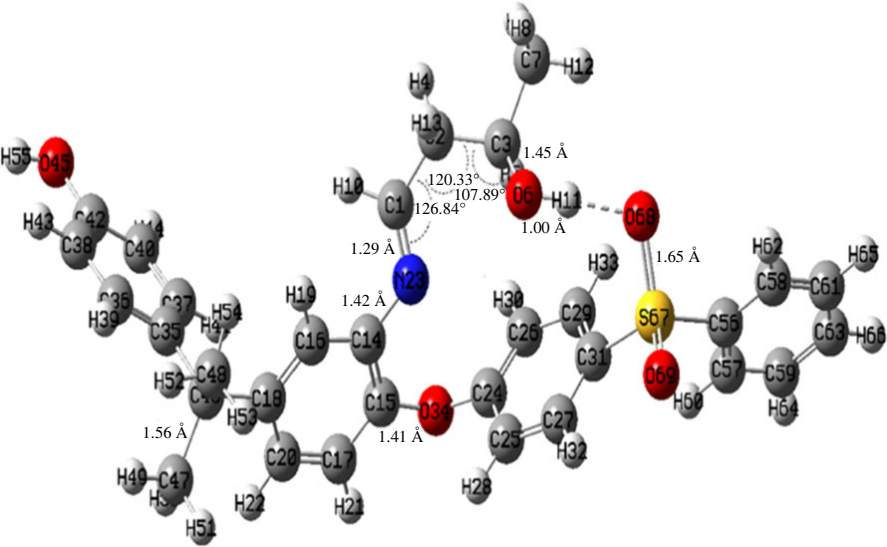
Optimized structure of PKI monomer. The hydrogen bond is represented by the dashed line [O(6)–H(11) · · · O(68)].

Quantum calculations were performed to confirm the PKI formation. The stationary points of the stable conformers of NH_2_-PES, OPVA and their synthesized PKI were investigated using the DFT/B3LYP-6–311 + G method. The optimized structures are shown in electronic supplementary material, figures S1, S2 and figure 3. The slight elongation of the O(6)–H(12) bond (0.98 Å) in the free OPVA (electronic supplementary material, figure S1) after condensation with H_2_N-PES to form Schiff base PKI (O(6)-H(11), 1.00 Å) suggested the formation of a hydrogen bond (O(6)-H(11) · · · O(68) (figure 5).

The electronic molecular parameters, including formation energy, frontier orbital energy and dipole moment, are summarized in electronic supplementary material, table S1. The lower total energy (*E*_T_) of PKI (−2028 arb. units) than that of the reactants (NH_2_-PES and OPVA) indicated that the direction of the reaction mechanism shifted toward the more stable product (PKI). Structural stability was also indicated by the calculated energy gap between the low unoccupied and highly occupied molecular orbitals (Δ*E* = *E*_LUMO_−*E*_HOMO_). Generally, a low energy gap suggests a low stability and high chemical reactivity. The Δ*E* of NH_2_-PES (3.962 eV) reflects its high reactivity and propensity to condense with OPVA, which matches the findings presented in figure 1.

The two frontier orbitals (HOMO and LUMO) of the investigated compounds are shown in electronic supplementary material, figures S3 and S4, respectively. A molecular electrostatic potential map (MEP) was constructed over the entire surface of OPVA, NH_2_-PES and PKI to identify the electrophilic and nucleophilic sites of the molecules (electronic supplementary material, figures S5 and S6).

#### 3.1.4. XRD and thermogravimetric analysis

**Figure 6.**
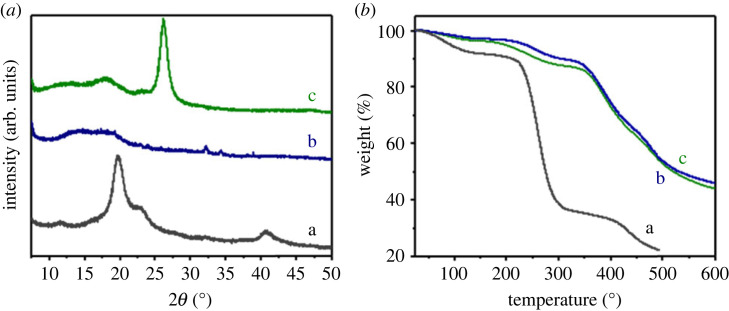
(*a*) X-ray diffraction patterns and (*b*) thermogravimetric analysis curves for (a**)** OPVA, (b) H_2_N-PES and (c) PKI.

Both starting polymers (OPVA and H_2_N-PES) showed peaks at 2*θ* values of 20°, 22°, 25° and 41°, indicating that these polymers were partially crystallized (figure 6*a*). The new polymers, with different percentages of OPVA, only displayed large ill-defined peaks in the 2*θ* range of 10–25°. Crystallinity was partially lost, probably because of the chemical reaction, which introduced new groups linked to the PES backbone that hindered the arrangement of the polymers into a crystallized form.

Owing to its nonaromatic nature, OPVA is less stable and more prone to thermal decomposition. Its mass decreased at approximately 100°C, probably because of the loss of residual water molecules or dehydration of the polymer (figure 6*a*). A substantial decrease in mass was also observed starting at 250°C, owing to the decomposition of OPVA. H_2_N-PES was more stable and showed two instances of mass loss at 200°C and 400°C. The second instance of mass loss was probably due to the decomposition of the polymer. The thermal stability of the new polymers was similar to that of the starting H_2_N-PES (figures 6*b* and 6*c*). This pattern was probably related to the low quantity of OPVA and the presence of an aromatic backbone, which provided thermal stability. All polymers (1 wt. %, 3 wt.% and 6 wt.% of OPVA) had a similar decomposition pattern up to 350°C; above this threshold, a slightly different decomposition behaviour was observed.

### 3.2. Characteristics of the laser-induced graphene electrodes

#### 3.2.1. HET rate

To further assess the intrinsic electrochemical activity of the laser-induced electrodes, we investigated the heterogeneous electrode transfer mechanism of the fabricated electrode using an equimolar mixture of [Fe(CN)_6_]^3–^ and [Fe(CN)_6_]^4–^ as a redox probe to investigate the electrode surface characteristics.

**Figure 7.**
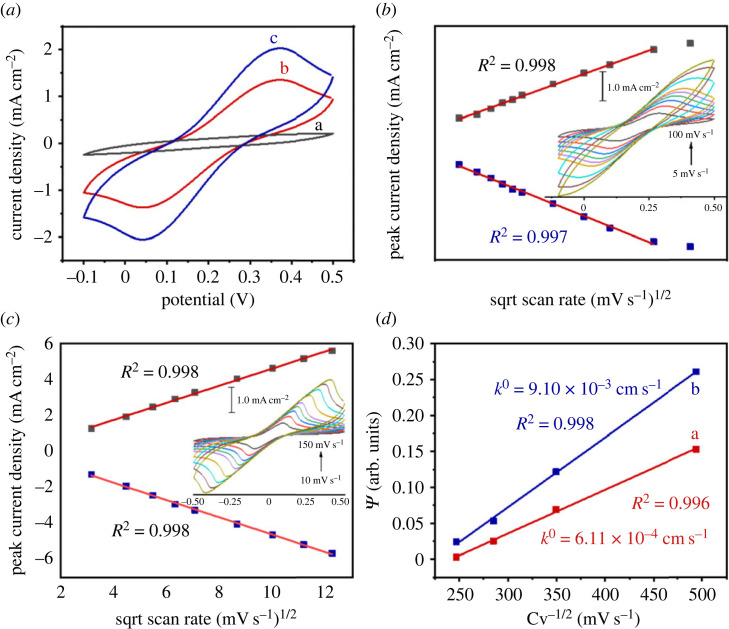
Electrochemical characterization of laser-induced graphene electrode. (*a*) CV in (a) a PBS solution or in a PBS solution containing 5.0 mM concentrations of [Fe(CN)_6_]^3–^ and [Fe(CN)_6_]^4–^ using a bare LIG electrode (b) or using an AuNPs-modified electrode (c) recorded at can rate of 50 mV s^–1^ (*b*) Plots of the anodic and cathodic currents versus the square root of the scan rate (inset shows CV of the bare electrode at different scan rates), (*c*,*b*) Plots of the anodic and cathodic currents versus the square root of the scan rate (inset shows CV of the AuNPs-modified electrode at different scan rates), and (*d*) plot of the *Ψ* function versus the inverse square root of the scan rate for the bare (a) and the AuNPs-modified LIG (b) electrodes.

Ferrocyanide [38–40], hexaammineruthenium(II)/(III) (Ru(NH_3_)_6_^2+^/^3+^), hydroquinone and other catechols [41,42] are generally used as inner sphere mediators to provide information on the electron transfer mechanisms at the electrode-solution interface. After the electrode was stabilized, it was examined in 0.1 M PBS solution. The CV curves of bare LIGE in a fresh solution of PBS did not show any peaks related to the oxidoreduction of the electrode material (figure 7*a*). However, two intense peaks related to the oxidation and reduction of the ferricyanide complex on the three-dimensional surface of the graphene electrode were observed in the presence of 5.0 mM concentrations of a redox probe solution. The peaks were symmetrical, and the current density was high (*J*_P_ = 1.30 mA cm^–2^), which indicated a high surface area and good catalytic properties of the laser-induced three-dimensional graphene network. The scan rate effect on the peak current showed a monotonous increase in the peak intensities with an increase in the scan rate (figure 7*b*). The plots of the peak current versus the square root of the scan rates were linear, which suggested that the transport of the reactants to the electrode was a diffusion-limited process (figure 7*c*). Additionally, adding gold nanoparticles to the electrode surface further improved its performance. Indeed, the current density of electrode increase by 62% (*J*_P_ = 2.12 mA cm^–2^), and the peak-to-peak potential separation decreased.

#### 3.2.2. Electrochemical active surface area

The intrinsic electrochemical behaviour of LIGE increased its suitability for electrochemical sensing applications because the three-dimensional porous graphitic network provided easy access to the electrolyte ions in the carbon-active material. Carbon was used as the quasi-reference electrode. First, the Randles–Sevcik equation (3.1), for quasi-reversible systems, was used to estimate the electrochemically active surface area (ECSA) of the LIGE and the AuNPs-modified one [42]. The ECSA was found to be 2.5 times and 4.2 times higher than the geometrical area (A_geo_) for the bare and AuNPs-modified electrodes, respectively. This observation highlights the advantageous effect of incorporating gold nanoparticles, which significantly enhance the electrode's three-dimensional surface area.

3.1 $$i_{p}=\pm 0.436\times \mathrm{nFA}\sqrt{\frac{nFDv}{RT}}.$$

Here, *A* is the electrode surface area (cm^2^), *D* is the diffusion coefficient of the molecule (6.70 × 10^−6^ cm^2^ s^−1^) [43], *n* is the number of electrons participating in the redox reaction, *C* is the concentration of the redox probe and *v* is the scan rate (V s^−1^).

Next, the Nicholson equation (3.2) was used to determine the heterogeneous electron transfer (HET) rate (*k*^0^) [44].

3.2 $$\Psi =k^{0}{\left(\frac{D_{0}}{D_{R}}\right)}^{\alpha /2}\sqrt{\frac{RT}{\pi nFD_{0}v}}=\;k^{0}\sqrt{\frac{RT}{\pi nFD_{0}v}}.$$

This equation can be simplified since the values of *D*_O_ and *D*_R_ are approximately equal and *α* is equal to 0.5. In the equation, *Ψ* indicates the dimensionless kinetic parameter, *k*^0^ indicates the HET rate, *D*_O_ and *D*_R_ indicate the diffusion coefficients of [Fe(CN)_6_]^3–^ and [Fe(CN)_6_]^4–^, respectively, *α* is the transfer coefficient, *n* indicates the number of electrons involved in the process (*n* = 1), *F* is the Faraday constant (*F* = 96. 489 C mol^−1^), *v* is the scan rate in (V s^−1^), R is the gas constant (8.314 J mol^−1^ K^−1^) and *T* is the absolute temperature (K). In this case, *Ψ* depends only on ΔE_p_, and can be determined using equation (3.3).

3.3 $$\Psi =\;\frac{\left(-0.6288+0.0021X\right)}{\left(1-0.017X\right)},$$

where *X* indicates the peak potential separation (in mV) multiplied by the number of electrons. Therefore, using equation (3.3), the HET rate can be determined from the slope of the *Ψ* versus the Cv^−1/2^ plot (figure 7*d*). We estimated the value to be 6.11 × 10^−4^ cm s^−1^, which was approximately one order of magnitude lower than that reported by Nayak *et al.* (11.5 × 10^−2^ cm s^−1^) [45], Griffiths *et al.* (2.37 × 10^−2^ cm s^−1^) for laser-induced GO reduction [46], Bosch-Navarro *et al.* (1.40 × 10^−2^ cm s^−1^) for CVD graphene [47] and Kavai *et al.* (3.20 × 10^−2^ cm s^−1^) [27]. We attributed this effect mainly to a slower electron transfer rate, as indicated by the large peak separation (≈ 300 mV), which was higher than that found in the studies cited above.

The performance of the HET was significantly enhanced through functionalization with gold nanoparticles. Remarkably, the HET rate experienced a 15-fold increase, soaring from 6.11 × 10^−4^ cm s^−1^ to 9.10 × 10^−3^ cm s^−1^.

### 3.3. Sensing of paracetamol

#### 3.3.1. Using bare LIGE

**Figure 8.**
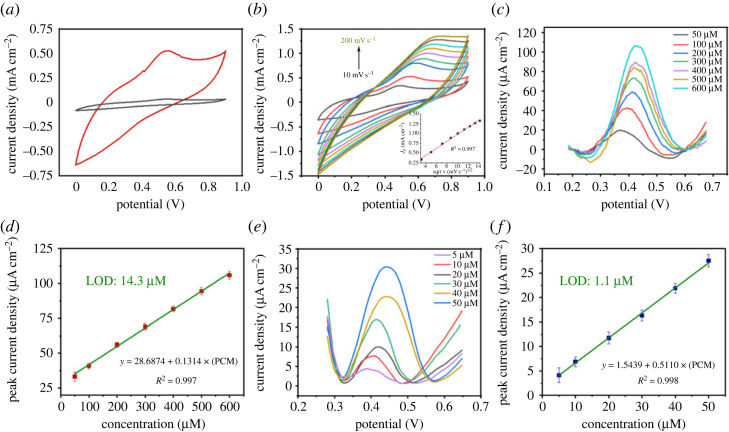
Electrochemical application of laser-induced graphene electrodes for paracetamol analysis. (*a*) Cyclic voltammetry in (a) PBS and (b) paracetamol (100 µM) dissolved in PBS 0.1 M. (*b*) Cyclic voltammetry at different scan rates of paracetamol solution (inset: plot of the anodic current versus the square root of the scan rate). (*c*) SWV curves of the bare LIG electrode in the presence of different concentrations of paracetamol. (*d*) Calibration curve of peak current density versus paracetamol concentration (50–600 µM). (*e*) SWV curves of the AuNPs-modified LIG electrode in the presence of different concentrations of paracetamol (5–5 µM). (*f*) Calibration curve of peak current density versus paracetamol concentration for AuNPs-modified electrode.

We first examined the effect of PCM on the prepared LIGE electrode. When the target molecule was absent, no electrochemical feature denoted that the electrode was electrochemically inactive, while in the presence of 100 µM solution, we detected two peaks, one around 0.45 V, which was related to the oxidation of PCM, and an ill-defined peak around 0.2 V, which was attributed to the reduction of the oxidized form (figure 8*a*).

The increase in the scan rate induced a monotonic increase in the oxidation peak current density, which depended on the square root of the scan rate, indicating that a diffusion-controlled electrochemical process occurred. This suggested that PCM did not adsorb or polymerize on the electrode surface, as reported by Ghanam *et al.* [26] (figure 8*b* and the inset in figure 8*b*). When the SVW parameters were optimal, an increase in the concentration of PCM from 50 µM to 1000 µM showed that the current density increased concomitantly with an increase in the concentration (figure 8*c*). Plotting the peak current density versus the PCM concentration showed linear behaviour in the range of 50–600 µM, which was determined using equation (3.4) (*R*^2^ = 0.997) (figure 8*d*).

3.4 $$j(\mu A/cm^{2})=28.6874+0.1314\times [\mathrm{PCM}\;(\mu M)].$$

The limit of detection was 14.3 µM, and the ratio of the standard current deviation of the five blank electrodes divided by the slope of the calibration plot. LIGE was able to detect PCM across a wide range of concentrations.

#### 3.3.2. Using AuNPs-LIGE

The functionalization of the electrode surface with gold nanoparticles resulted in increased sensitivity towards PCM. Indeed, the modified electrode exhibited a linear relationship between peak current density and PCM concentration within the range of 5–50 µM, as described by equation (3.5) with a high coefficient of determination (*R*^2^ = 0.998) (figure 8*e* and *f*). The equation relating the peak current density (*j*) to the paracetamol concentration (PCM) is given as

3.5 $$j(\mu A/cm^{2})=1.5439+0.5110\times [\mathrm{PCM}\;(\mu M)].$$

Furthermore, employing the same method, the detection limit for PCM was determined to be 1.1 µM. It is worth noting that the AuNPs-modified electrode exhibits higher sensitivity to PCM compared to the bare electrode, as evidenced by the slope of the linear relationship being approximately 3.9 times greater (figures 8*d* and 8*f*). This enhanced sensitivity can be attributed to the incorporation of gold nanoclusters through the functionalization process.

### 3.4. Performance of LIGE

#### 3.4.1. Stability

**Figure 9.**
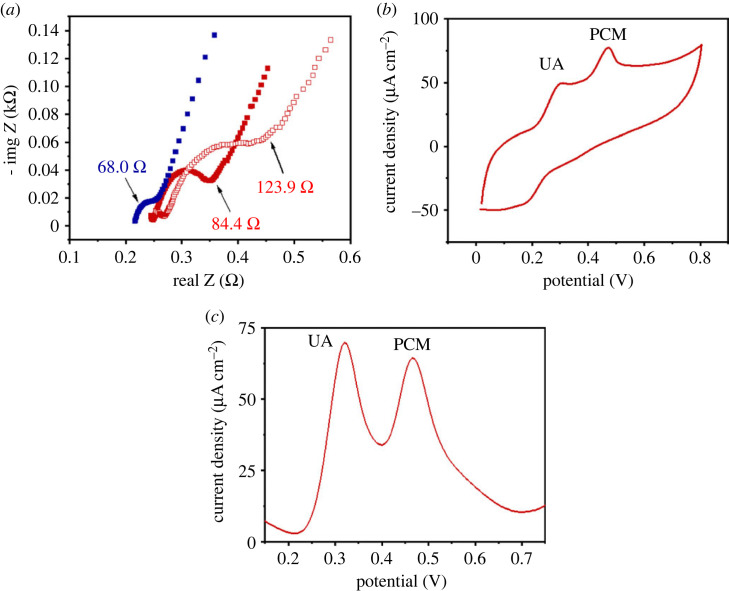
Electrochemical stability and selectivity of the LIG electrodes. (*a*) Nyquist plots of bare electrode (a), AuNPs-modified electrode (b) and the bare electrode after 1000 successive CV cycling. (*b*) Cyclic voltammetry and (*c*) SWV curves of UA (500 µM) and PCM (500 µM) dissolved in PBS.

The addition of the gold nanoparticles improved the electrode conductivity. Indeed, the initial charge-transfer resistance (R_CT_) was 84.4 Ω, which decreased by 19.4% (68.0 Ω), showing the beneficial role of functionalization with gold particles (figure 9*a*). Next, we assessed the stability of the electrode by recording the Nyquist plots before and after 1000 consecutive CV cycles. The R_CT_ (84.4 Ω) increased to 123.9 Ω after cycling (figure 9*a*). This increase of 31.8% indicates that the electrode remained relatively stable and could be reused. In addition, these electrodes are inexpensive since they prepared from low-cost polymers and can be discarded after a single analysis.

#### 3.4.2. Selectivity

The selectivity of the electrodes was assessed by recording the cyclic voltammogram in the presence of UA, which has an oxidation potential peak close to that of PCM (figure 9*b* and *c*). The CV showed two distinct oxidation peaks at *ca* 0.3 V and 0.5 V, respectively, attributed to UA and PCM. This was confirmed by SVW voltametric analysis, which showed two non-overlapping peaks at 0.32 V and 0.48 V, thus the PCM could be determined from drug samples containing both compounds with interference.

### 3.5. Comparison with previous studies

We compared the performance of the electrodes used in this study with those reported in other studies for the electrochemical sensing of PCM. The data are presented in table 1. Furthermore, we also considered the electrode functionalized with gold nanoparticles.

**Table 1.** Performance of electrodes used for the electrochemical sensing of paracetamol. AuNPs: gold nanoparticles; LIGE (LSGE): laser-induced (-scribed) electrode; PKI, polyketimine; PEI, polyetherimide; MWCNTs, multi-walled carbon nanotubes; PANI, polyaniline; PAMAM: poly amido amidine dendrimer; pGly, poly glycine; nano MIPs, nanometric molecularly imprinted polymers; ITO, indium-doped tin oxide; rGO, reduced graphene oxide; RuO_2_: ruthenium (IV) oxide; CB, carbon black; CPE, carbon pencil electrodes.

electrode	method	dynamic range (µM)	detection limit (µM)	ref.
LIGE on PKI	SWV	50–600	14.3	this work
AuNPs-LIGE on PKI	SWV	5–50	1.1	this work
LSGE on PI	SWV	0.1–10.0	0.004	[25]
LIGE on PI	SWV	0.1–0.01	0.031	[26]
LIGE on PEI	SWV	0.0–50	0.323	[27]
MWCNTs/PANI/LIGE on PI	SWV	0.1–55	0.006	[28]
MWCNTs/PAMAM/GCE	DPV	0.3–200	0.1	[48]
MWCNTs/pGly/GCE	DPV	0.5–100	1.7	[49]
RuO_2_/Nafion/GCE	SWV	5–250	1.2	[50]
NanoMIPs/SPCE	DPV	100–1000	50	[51]
nAu/ITO	DPV	0.2–1500	0.18	[52]
rGO/GCE	DPV	0.005–800	0.002	[53]
rGO/CB/SPCE	SWV	10–200	1.5	[54]
CPE	LSV	12.4–200	3.7	[55]

As shown in table 1, our sensor had a wider dynamic range than sensors in most studies [24,27,51–53]. However, the range was narrower than those of sensors that used nanoMIPs/SPCE [51], nanogold on ITO [52] or reduced graphene oxide on top of glassy carbon electrodes [53,54]. Our detection limit is higher than that of the sensors listed in table 1. The other sensors had detection limits at the micromolar level due to the use of nanomaterials, such as MWCNTs, rGO, Ru_2_O nanoparticles and nanogolds, which lowered the limit of detection. Adding gold nanoparticles allowed detecting lower concentrations of PCM and improved the detection limit by one order of magnitude from 14.3 µM to 1.1 µM, which become comparable to those using other nanomaterials.

## 4. Conclusion

We synthesized a PKI membrane via the chemical condensation of OPVA and NH_2_-PES, which was characterized using several physico-chemical analysis techniques and DFT. The membrane was used to scribe graphene electrodes using a CO_2_ laser beam. LIGEs were characterized using electrochemical techniques to show that they had a large surface area and HET rate, which were improved by adding gold nanoparticles to the electrode surface. The LIG electrode can be used for sensing PCM in spiked buffered solutions in the linear range of 50–600 µM with a detection limit of 14.3 µM. Furthermore, functionalization of the electrodes with AuNPs allowed the sensing of PCM at lower concentration (5–50 µM) and increased the limit of detection to 1.1 µM. Thus, it is a promising candidate for sensing other drugs, pesticides and other small organic molecules. They may also be integrated into microfluidic devices or wearable sensors.

## Data Availability

Our data are deposited at the Dryad Digital Repository: https://doi.org/10.5061/dryad.sbcc2frc8 [56]. The data are provided in electronic supplementary material [57].
